# The Depathologising University

**DOI:** 10.16993/sjdr.1240

**Published:** 2025-03-19

**Authors:** Dan Goodley, Kirsty Liddiard, Rebecca Lawthom

**Affiliations:** iHuman, School of Education, University of Sheffield, UK

**Keywords:** depathologising, university, research project, critical disability studies, research culture

## Abstract

It has been argued that the university needs depathologising, a radical rethink and reorientation of the university’s relationship with disability. This paper offers an original affirmative proposition: that the university is already depathologising. Inspired by disabled people’s activism and scholarship, we explore the ways in which academics, researchers and research professional colleagues are depathologising the disablist and ableist university. We reflect on our practices as principal investigators and research leaders of three funded research projects using novel composite conversations (a unique methodological form of experimental writing) and explore (i) pushing back at university bureaucracy towards co-production; (ii) critically appropriating the performative university and (iii) enabling access as colleagues. Depathologisation invites us to pause, to meditate and to significantly reimagine the university. And those of us who work in the university are the university, and we all have work to do.

## Introduction

In a recent paper, one of the authors developed a critical disability studies conversation with decolonisation to pitch a novel mode of engagement: depathologising the university (Goodley 2024a). Depathologisation sits with the lessons of decolonisation to desire disability’s disruption of the university. Decolonisation deconstructs racist legacies of colonialism, racism, white supremacy and Western European privilege (Zondi 2022). Depathologisation deconstructs the disabling legacies of ableism, an ideology that privileges able-bodiedness and -mindedness and a preferential citizen as self-sufficient, autonomous, independent and entrepreneurial. Ableism feeds disablism, which is discrimination against people with physical, sensory, cognitive impairments and neurodivergence, because of ableism’s preference for those deemed to be abled (Wolbring 2008). We do not conflate ableism and colonialism nor assume that ability and white privilege are equivalent phenomena. Instead, we understand depathologisation as a counterpoint to ableism. Much can be learnt by bringing together decolonisation and depathologisation to converse with one another to rethink the university (Goodley 2024a).

The university is an ableist institution. Disabled students and staff experience exclusionary admissions and recruitment, poor career pipelines and in/formal support, under-employment and precarity. Disabled and neurodivergent academics and researchers contest everyday forms of ableism and disablism in the academy (see Gill 2009; Shah 2018; Olsen et al. 2020). The university is also, to varying degrees, an elite institution. Those from wealth and privilege tend to flourish because the academy has been designed with them in mind. The individual human being at the heart of ableism befits the model of the insular citizen instituted by western, liberal democracies. Universities are key institutional players in these democracies and work as sorting machines, inviting in, educating, grading, promoting or rejecting students and staff on the basis of performance. Academics, researchers, technical and professional services colleagues are caught up in this institutional machinery. For disabled people—those with physical, sensory or cognitive impairments and neurodivergence—these normative practices are exacerbated by the impacts of disablism, everyday forms of discrimination and marginalisation experienced by disabled people (Thomas 2007). Disablism is a by-product of ableism, neoliberalism and capitalism, where competitive individuals are cherished and disabled people are routinely conceptualised as deficient.

When a university moves to the beats of ableism and the rhythms of disablism, then this clashes with the aspirations of disabled people. We find hope in two fields of inquiry. Critical disability studies is an interdisciplinary field of scholarship and activism where disability is constituted as a socially, culturally and politically constructed phenomenon and recast in terms of possibility, radicality and affirmation (McRuer 2006; Meekosha and Shuttleworth 2009; Titchkosky 2011; Shildrick 2012). Disability drives a rethink of how we understand and work with one another in the university. Decolonial studies pulls together theoretical and political alliances from Black, Indigenous, and People of Colour (BIPOC) (Wynter 2003; McKittrick 2015; Zondi 2022), overturning practices of white privilege to reimagine the university. Just as decolonisation draws upon radical scholarship of BIPOC, then depathologisation draws on the contributions of disabled people. The promise of depathologising the university is found in its re-humanisation of the institute, a rebuilding of inclusive communities, the mutual recognition of others, an embracing of our relational selves, a requirement for intersectional engagement, a commitment to epistemic justice and equitable collaborations with disabled people’s organisations (Goodley 2024a). Disability is also radically reconceptualised, from a pathological problem in need of fixing to a driving subject that leads a recalibration of the university.

We argue that the university is already a depathologising university. Thinking with the decolonising writing of la paperson (2017), we focus on research projects to explore some affirmative ways in which academics, professional services colleagues, disabled people’s organisations and researchers are depathologising the disablist/ableist university. As we unpack these acts and encounters, we rub up against the performative and bureaucratic machinations of the university. La paperson’s *A Third University Is Possible* is a commentary on ‘building a decolonising machine out of colonising scraps’ (la paperson 2017, 51), working with ‘the multiple flows of command and various vertical, hierarchical, lateral and organic codes’. An effective decolonising university shares ‘a love for Black life, for Indigenous worldings, for their futures’ (la paperson 2017, 52) and already exists because it ‘is assembling, is strategic, is timely (though its usefulness constantly expires), is unromantic (and not worthy of your romance), is problematic (in all likelihood, it charges fees and grants degrees), is anti-utopian and is a machine that produces machines’ (la paperson 2017, 53). These words resonate with us as we think about the depathologising university.

Sitting with a depathologising university reveals a parallel reality: a pathological and colonial university system where disabled and BIPOC colleagues are often not imagined to be present nor participating. And yet, in these contemporary times, we also find a desire for disability in the university as more and more people identify with disability and neurodivergence. Similarly:

‘…within the colonizing university also exists a decolonising education. Occupying the same space and time are the coloniser’s territories and institutions and colonised time but also Indigenous land and life before and beyond occupation’ (la paperson 2027, xiii)

La paperson (2017, 44) refuses to embrace a utopic description of decolonisation, instead appealing to those of us ‘involved in university projects with decolonial desires to implement change pragmatically’ and others who have ‘appropriated university resources to synthesise a transformative, radical project’. There is always friction: ‘many gears rubbing against each other – bodies, literally, rubbing against each other …through these associations of rubbings, frictions, and greasing of gears, new formations come into play’ (la paperson 2017, 54). The depathologising university also enacts a frictional politics, a form of analytics and community building that rub up against one another, as la paperson notes, creating uncomfortable conversations, lines of flight, unexpected practices, conversations, concepts and desires.

Disability is a quintessential frictional subject. Categories of disability offer ‘impasse and possibility’ (Karakilic 2019, 499), simultaneously signifying lack (hegemonically prescribed as the antithesis of ability) and possibility (as articulated by disabled activists, artists, researchers and theorists who demand to be known in terms of plenitude and abundance). Critical disability studies herald the deconstruction of pathologisation alongside the celebration of disability’s potential (see McRuer 2006; Meekosha and Shuttleworth 2009; Titchkosky 2011; Shildrick 2012; Goodley 2024b). Disability is ‘both a mark of the most endemic control’ (framing people solely in terms of a category) and ‘a sign of a new insurgency’ (offering framings that can be used for various disruptive and empowering means) (Toscano 2007, 112). When disability is rendered deficient, it calls forth a host of pathological, curative and rehabilitative discourses. When disability is understood as opportunity, it provides new affirmative ways of thinking about and being together in the university. This feels like Braidotti’s (2019a; 2019b) concept of affirmative ethics: the philosophical, methodological and political project of affirming the possibility of a here and now that is liveable and sustainable.

We write as principal and co-investigators of research projects to pause and reflect on what it means to lead, coordinate and manage. While people are always the key elements of research, it is sobering to note how quickly people morph into bureaucratic cogs, systemic mechanisms and policy discourses of universities and funders. The ‘performative university’ reproduces a state of affairs ‘where the transmission of knowledge has increasingly become attuned to the needs of business and society as a form of ‘mercantilization of knowledge’ (Lyotard 1984, 51; in Jones et al. 2020, 365). We explore some of the challenges of promoting disability research and building an inclusive research culture in the performative university. As a mix of white, cis-gendered, straight, disabled and non-disabled academics, first- and second-generation university graduates tasked with university leadership responsibilities, our positions often feel impossible. la paperson (2017, xxiii) writes:

My position is impossible; a colonialist-by-product of empire, with decolonising desires. I am, and maybe you are too, a produced colonialist. I am also a by-product of colonisation. As a colonialist scrap, I desire against the assemblage that made me’ (le paperson 2017, xxiii).

It is incumbent on us to associate ourselves with scholarly and activist assemblages that coalesce around disability politics (Waitt et al. 2023) to move from disability-as-problem to disability-as-possibility. Our work recentres disability as the subject through which to think generatively about the university.

## Methodology

We reflect on our roles as principal/co-investigators of three externally funded projects. *Disability Matters* is a six-year research programme funded by a Wellcome Trust Discretionary Award that seeks to develop anti-ableist and anti-disablist approaches to scholarship; broadening health research priorities, innovating research methodologies, promoting inclusive research environments, encouraging more positive disability representations and building a new generation of disabled and disability-focused health researchers. Our programme aims to generate transformative equity, diversity and inclusion knowledge to challenge ableism and disablism in the practices and cultures of health research. We work in collaboration with Disabled People’s Organisations (DPOs) from around the world.

*Humanising Healthcare* is a three-year research programme funded by the Economic and Social Research Council that deploys a co-production framework of co-leadership and management of a qualitative study of a neurology service and a learning disability service. We seek to identify forms of humanising, compassionate, person-centred and empowering healthcare practice enacted by healthcare professionals with patients with learning disabilities.^1^ Key to this project and relevant to this paper is the central role of researchers with learning disabilities who draw upon their personal experiences of healthcare and the research and innovation of their advocacy-based organisations. These organisations—Barod, Sheffield Voices, Sunderland People First and Speakup Self-advocacy—are members of our executive research team and are tasked with helping deliver the project from inception to completion.

### Cripping Breath

Towards a new cultural politics of respiration is a five-year transdisciplinary programme of research funded by a Wellcome Discovery Award. It centres and explores the lives of people who have had their lives saved and sustained by ventilatory medical technologies. Centring arts-informed, archival, narrative and ethnographic approaches, Cripping Breath develops Crip perspectives, forms of knowledge production that emerge from lived experiences of disability and chronic illness. Academics, researchers, experts-by-experience, clinicians and artists are working in collaboration to co-curate and co-produce new understandings of the experiences of ventilated people across a host of identity positions to interrogate the new cultural politics of respiration and ventilation in a continuing global pandemic and as we imagine post-pandemic futures. Key to our co-produced approach is the Community Researcher Cooperative, a team of 13 community-based researchers, all of whom live on and with ventilatory technologies and respiratory illness, working across the project to embed lived and embodied knowledge into our theory-building and co-lead our inclusive approaches to inquiry (see Liddiard et al. 2024; Liddiard et al. 2022).

While all three projects have received ethical clearance, this paper does not use their data. Instead, we critically interrogate some of our practices as project leaders. Our focus upon research projects is deliberate. Rather than conceptualising the research projects as an arbitrary container, we understand each project as:

‘a cultural and political sorting mechanism, used to divide and stratify people according to task. Some tasks proliferate in project time, particularly structurally invisible tasks associated with administration, finance and documentation, while others narrow in scope, such as what counts as an inquiry or research outcome’ (Viney 2024).

The research project evokes particular forms of governance, labour and accountability, offering an entry point into critical explorations of the depathologising university. Our three projects include the authors, 12 co-investigators (from different universities and disciplines, including nine academics and three clinical researchers), eight researchers (the majority of whom are early-career researchers), 10 DPOs offering strategic leadership as paid non-academic partners, one project manager and a programme manager (both members of professional services staff), 13 community researchers (people with lived experience of disability and chronic illness who bring embodied and other forms of expertise to the research), three hospital trusts, one arts organisation, two artists-in-residence and six disabled and chronically ill artists.

Our projects reflect the cultural turn towards Equality, Diversity and Inclusion (EDI) within university, research and funding sectors (Lett et al. 2022). Research funders continue to place a premium on high-quality discovery research but also emphasise the need to promote diverse research cultures and proactively develop the careers of researchers and professional services colleagues. Work on decolonising and depathologising the university has become more visible in recent years (Bhambra et al. 2020; Brown and Leigh 2020; Housee 2022; Goodley 2024a). What this means in terms of practice, however, is often unclear, with some commentators worrying that universities remain rhetorically responsive rather than pragmatically engaged (Ball 2022). Furthermore, EDI and DEI (Diversity, Equity and Inclusion as it is framed in North America) face a major contemporary backlash (Hamilton 2024; Walk-Morris 2024). We want to acknowledge the fear and distress that many of our colleagues are experiencing within the university.

Our orientation to the writing of this paper is one in conversation with la paperson’s (2017) provocation that universities are already engaged in the process of decolonising the university. We extend this metaphor to depathologisation with the following ambition: to capture those moments of disruption, transformation and change offered by the presence of disability in the university via the research project.

While we turn to the research project as a cultural site through which to contemplate the depathologising university, we do not directly draw on the projects as data. Instead, we use our projects as springboards from which to consider our roles as research leads. Our methodology draws on some common themes, broad strokes, moments of contemplation, critical reflections and stories that emerge from our responsibilities as project leaders. Narrative has been widely adopted as a methodology for capturing experiences and perspectives of academics and student leaders from BIPOC and disability communities (Hotchkins and Dancy 2015; Luna 2022; Williams et al. 2022). Key writers within these communities remind us of the productive nature of storytelling. For example, disability studies theorists tell us that we construct theory through narrative (Garland-Thomson 2014) and that stories permit us to think of things anew (Michalko 2017). As Thomas King (2003, 2) writes, ‘the truth about stories is that that’s all we are’. And so we turn to our own storied conversations about our attempts to promote inclusive research cultures, proactively build the careers of our research colleagues and work collaboratively with key disability organisations.

As co-authors, we spend a lot of time telling stories during regular online and offline tête-à-têtes. Viney (2024) writes that, while it is common to find critical dialogues around ‘disciplinary hierarchies, epistemological differences, interpersonal and individualising clashes around conflicting work ethics and professional conduct—rarely is the project held accountable’. We seek to capture the flavour of some of our project lead conversations with one another, ever mindful of not exposing the specifics of what we are talking about. While conversations constitute a common data set for qualitative researchers (e.g., Alexander et al. 2024), ‘they are an under-used and rather unsung method in qualitative, social research’ (Swain and King 2022, 8). We adopt the writing of composite conversations, inspired by the composite narrative method, which uses data from several data sources to tell a single story (Lawthom and Kagan 2016; Willis 2019). Composite conversations draw on regular discussions between the authors, reflecting upon project work and its place in the university. Our conversations, presented below and italicised, were constructed from a collective review of our email correspondence, face-to-face and phone discussions as we grappled with our attempts to depathologise the university. We have chosen to deploy three composite characters: Brian, Ann and Eve. This approach has freed up our writing. Elements of the conversations represented in this paper can be traced back to our actual conversations as well as debates and dialogue we have had with other academics, project investigators, researchers and professional services colleagues over a number of years of working in the university. Our composite conversationalists, Brian, Ann and Eve, embody the feel of some of our discussions with one another and a myriad of colleagues that ultimately inform the key analytical desire of this paper, which is to evidence a depathologisation of the university. Their conversations are best read not as direct reflections on the three foundational projects of this paper but as loose fabrications informed by the authors’ conversations as we reflect on our roles as Principal Investigators. Composite conversations raise methodological questions about truth, rigour and authenticity. There are very real stories at the heart of our composites. We build on a long tradition of educational research that has embraced experimental and narrative approaches in qualitative research and made a strong case for the rigorous nature of storytelling and its opportunities for creating insightful data (e.g., Bolton 1994; Clough 2000; Spindler 2008; Piper and Sikes 2010).

## Analysis

Our analysis turns to three themes reflecting our growing understanding of depathologisation and products of inductive and deductive analysis as we move between theoretical ideas, our own notes and the draughting of composite conversations. We were encouraged by Spindler (2008, 28) to embrace experimental narrative writing that has ‘the potential to resonate with practitioners so that they discover new ways of thinking and feeling about professional dilemmas that go beyond ‘mere truisms’ to a deeper understanding of the significance of their professional actions’. Our imagined audience includes colleagues working within universities, many of whom might be already practising depathologisation.

### (I) Pushing Back At University Bureaucracy Towards Co-Production

In working collaboratively with DPOs, we have witnessed the rich possibilities for co-creating philosophical, methodological and analytical approaches to research. We have published together in a variety of formats (e.g., Bottomley et al. 2024; Liddiard et al. 2022; Liddiard et al. 2024) and remunerate DPOs through the payment of competitive consultancy rates to offer strategic leadership, analytical input and share our work through their networks. Our collaboration offers a discrete depathologising pathway to impact within the university, centring the knowledge of DPOs within the academy to trouble and transform theory, methodology and analysis. Over the three projects, we have budgeted £750,000 to pay DPOs. This triggers particular kinds of labour within the university.

Thomas King writes in his 2014 novel, *The Back of the Turtle*, of a First Nations community returning to their homeland, a cherished place that has been devastated by White settler extraction and pollution:

‘A boat runs ashore on the beach of the community and two characters debate pushing the vessel back into the ocean. When are you going to get another chance to push a ship off a beach? Mara asks of Gabriel. You’re never going to move it, complains Gabriel “It’s not about the moving”, says Mara, “It’s about community”‘ (King 2014, 498).

Recent scholarship has affirmed the perils of romanticising co-production. Oliver et al. (2019, 1) ‘advise a cautious approach to co-production’ based on such little empirical evidence of ‘how coproduction changes research, policy or practice, or how it may compare to alternatives’ (ibid. 2). Similarly, Williams et al. (2020, 1) argue that researchers who adopt participatory approaches should ‘advocate for greater consideration of the structural inequalities in academia and beyond that impede co-production’. King’s words resonate with us as we push against university bureaucracy to bring in the involvement of our DPOs, which, simultaneously, creates community. Titchkosky (2020, 198) encourages us to explore disability’s bureaucratisation in our universities, precisely because this helps us to learn something about the organising force of bureaucracy in all our lives. This bureaucracy continues as we develop collaborative relationships with our DPOs.

Eve: I’m proud of our working relationships with DPOs but minded of the barriers created by the sheer force of university bureaucracy’s impact on these relationships.

Brian: I couldn’t agree more. For money to flow from funder to university, to DPO this pulls into the administrative machinery of the university. And this is where, to follow King, we do some pushing back!

Ann: We rely on professional services colleagues; many of whom are over-worked, over-busy and under-represented. Collaborative agreements are written up with DPOs that contain impenetrable legalese. Due diligence checks are demanded. DPOs have to demonstrate that they are trustworthy organisations through the sharing of a plethora of documents that are subjected to audit. The setting up of vendors on university systems. The endless to and for of emails. The triggering of and waiting on payment of invoices.

Eve: We try to push back at the esoteric language of bureaucracy; to make things more understandable, practical, doable. We work closely with DPOs to support them through the processes. We chat online and meet in person. We share frustrations. We seek to explain the hidden labours that often get lost in the formal language of contract. On reflection we wonder if we are pushing back at all.

Ann: I hope we are creating community; working relationships between the university and advocacy-based organisations.

Brian: Our only hope is to hang on to each other! Co-production is our core business but university bureaucracies straightjacket expectations, aspirations and practical outcomes. We are bending and pushing bureaucracy.

The British university’s emphasis on grant capture engenders particular performative measures of success. Loureiro (2024) highlights the ‘endless array of metrics imposed by research funding agencies to assess the so-called “quality” and “return on investment” of our research’ and ‘an endless cycle of bureaucracy to prove we meet these arbitrary criteria—before, during, and … after we acquire funding’. There is an overwhelming sense of relief, success and celebration when a grant lands, but as William Viney (2024) observes, research projects pull us into administration, organisational hierarchical structures, inequitable relationships and debilitating modes of accountability. Political aspirations for and commitments to research often feel blunted as we are pulled into labour associated with financial and institutional administration. Holding these competing demands is often uncomfortable.

Eve: We find ourselves engaging with a truly ironic situation: how do we find the time to truly commit to research projects when we are consistently being pulled back into the administrative demands of the university?

Brian: I’m old enough to remember disabled researchers called for emancipatory disability research in the 1990s. Perhaps things are better now but still precarious; especially research of a co-produced and participatory persuasion.

Ann: The current clamour for funded co-produced research raises the question: what university administrative mechanisms are called upon when this work lands in the institution? While in the past it was right to bemoan the lack of support for such work, at least this work happened in the cracks, fissures and gaps of support. Now, as university’s embrace collaboration and co-produced inquiry, there is a danger of institutionalising and bureaucratizing this work.

Bureaucracy is deeply embedded in the colonialist and ableist histories of universities and might be read as a barrier to depathologisation. When disability rocks up at the university, it oftentimes becomes known as a problem to be solved by bureaucracy. Attempts to bring DPOs into the university to collaborate also call forth bureaucratic responses that sap energy and pull us away from the actual practice of research. And yet, it is incumbent on us to engage in these administrative processes to ensure that research projects are collaborative rather than institutionalised within the university. Working with university bureaucracy might be read as a ‘grudging act … activities in which we really would rather not participate but which we perform nonetheless’ (Bottero 2023, 533). ‘Such acts’, Bottero reminds us ‘play a significant role in how many social practices are routinely sustained, but also reworked or undermined’ (Ibid.). Bending, oiling and appropriating bureaucracy, alongside pushing back, feed depathologisation where disability drives research collaboration towards more emancipatory and inclusive models of disability research.

Brian: DPOs are research leaders, critical friends, auditors and theoretical provocateurs that hold research to account.

Ann: We should rethink our advisory board and research management groups as spaces where DPOs demand systemic change across the university sector.

Eve: There are numerous pragmatic and transformational actions that have been led by DPOs from informing the process of actively recruiting disabled and chronically ill researchers, to co-producing research questions and developing methods through to the rewriting of research outputs in a host of accessible formats.

### (II) Critically Appropriating The Performative University

While our research projects feel faithful to disability politics (where disability is the centre of our attention), the university feels like a more striating place (where disability is often placed on the periphery of the institution). We can find moments of depathologisation within this conflict. The university is a neoliberal-ableist university (Goodley 2024b) where the ideology of ableism is infused with neoliberalism: ‘the intrusion of an economistic logic into the academic field that expects universities to function and be managed like corporations to increase their productivity and competitiveness’ (Rogler 2019, 63). This transformation, Rogler argues, ‘confronts academics with a sharp increase in the competition for funding and the managerial control over their work, their working conditions becoming increasingly characterised by performance pressure and precarious employment’ (Ibid.). In Britain, academics and professional services colleagues are drawn into various complex and seemingly never-ending modes of accountability, assessment and surveillance associated with the TEF (Teaching Excellence Framework), REF (Research Excellence Framework) and KEF (Knowledge Exchange Framework), as well as a myriad of institutional policies, plans, strategies and visions.

Ann: I dare not count the hours a week we each spend in meetings, compiling documents or writing strategies that respond to these forms of assessment.

Eve: Our commitment to our researchers is driven by a desire to pass on security and permanence to others. This inevitably pulls us into form-filling and accountability creating further modes of enactment associated with the neoliberal university. We do recognise the privilege of working in a university in permanent posts; but this privilege comes at a price; an entanglement with complex, repetitive and continuous forms of assessment and accountability.

Brian: We need to resist our support of researchers as being yet another diary entry in a week of daily 8–6pm back-to-back meetings.

We know that colleagues in different contexts around the world have their own bureaucratic demands. We acknowledge a sense of dread and hopelessness when research risks being bureaucratically stymied. The British university’s emphasis on the importance of grant capture creates further forms of endurance and self-critique.

Ann: We are not seeking understanding nor sympathy from our colleagues; we are very much aware of the privilege of having the roles that we now occupy.

Eve: We need to be open, honest and reflexive about the debilitating and disabling impacts and cruel optimisms of the neoliberal-ableist university on the whole community, including ourselves.

Brian: Before our research grants finally landed we experienced failure, after failure, after failure. A litany of rejected grants. Unspoken periods of career nosediving.

We find ourselves engaging with a truly ironic situation: how do we find the time to truly commit to working with exciting projects when we are consistently being entangled with the bureaucratic demands of the neoliberal-ableist university? One answer resides in the support, mentoring and capacity building of the researchers and project managers involved in our projects. We are committed to building a new cadre of disability-positive and disabled researchers. This process begins with finding forms of inclusive recruitment that bring disabled researchers into academia (see Goodley et al. 2024 for a discussion) and continues with finding innovative, accessible and supportive forms of mentoring and capacity building. Commitment to early career researchers is now firmly institutionalised within the mission statements of universities and funders, as evidenced by The Concordat to Support the Career Development of Researchers (Vitae 2019).

Brian: The Concordat expects us to give 10 days per year to the career development of career researchers. We’ve gone further to allot one day per week; to really support our researchers to advance their careers.

Eve: As someone who has been in precarious contracts in the not-too-long ago past, there’s a sense of solidarity in meaningfully committing to mentoring;- a deep commitment to the person, as well as the career.

Ann: How can we expand the message of the Concordat to our research professional colleagues? They are absolutely central to our projects.

The Concordat encourages universities to think proactively about the capacity building of their researchers. This is often framed in performative measures of annual reviews, probation periods and continuing professional development courses. We are reminded of la paperson’s (2017, 6) comments that ‘colonising mechanisms are evolving into new forms, and they might be subverted toward decolonising operations’. Indeed, some ‘colonising technologies have been hotwired’ (Ibid., 23) in ways that aspire to truly develop the careers and aspirations of our colleagues.

Ann: I try to use Annual Reviews as an opportunity for researchers to reflect not only on their project work but also to sit with a sense of their own scholarship and their research ambition. This is especially important for disabled researchers because they may not have imagined to be part of the university.

Eve: It is really important to remind researchers of their own careers, writing passions and activist commitments because these can get lost in service to the research project. They are often on top of the key developments and debates in the field.

Brian: These conversations are crucial, especially when researchers move from doctoral to postdoctoral positions. We need to create opportunities for leadership, to centralise their scholarship and put in packages of support for career development from the moment they start to work on a project.

Critically appropriating a university’s technologies and performative measures of career development is especially important for disabled colleagues whose intimate and professional lives have been afflicted by ableism (Liddiard 2018; Brown and Leigh 2020). A core element of this work relates to addressing deep histories of exclusion on the part of disabled people in the university. A depathologising approach to mentoring and support contests the disavowal of disabled people and reminds one another that the university feels out of bounds for many folk.

### (III) Enabling Access As Colleague

Depathologisation centres disability as authority. Repositioning disability as the driving subject of the work of the university connects with arguments made by critical disability studies writers, such as Titchkosky (2011), Liddiard (2018) and Price (2024), who recast disability as wonder, possibility, opportunity and potentiality. Too often, access is understood in terms of the demands of an individual disposition, addressed as an institutional problem or written into a proposed policy that seeks to fix that problem. Instead, following Ruby Goodley (2025), we consider access as a colleague, a key member of our communities that turns up to the university offering possibilities but also difficult questions of how we might best work together.

Brian: Access as a colleague makes me think of other colleagues – academics, researchers, professional services, DPO colleagues – and how they are key to building research culture.

Eve: When I review the online space or the physical table of the face-to-face meeting, I survey the different kinds of colleagues who are present. Each colleague invites forms of support, capacity building, career development; enablement. And access works with other colleagues towards a new way of doing the university.

Ann: When we invite introductions from our members we have to make sure that all of our colleagues are there! Researchers? Here! Principal Investigators? Here? Project Managers? Here! Access? Here!

Brian: Access is not a distraction. Access is not a hindrance. Access is not a parallel process. Access is a welcome comrade.

Access joins other key colleagues to build research university research environments, working alongside researchers, academics, technicians, professional services, support staff, professionals, managers and project coordinators. These latter non-academic professional services roles are oftentimes othered and devalued, but their importance in building research environments cannot be underestimated (Caldwell 2024). As we work collaboratively with research and professional services colleagues, we find access to be another valued colleague.

Eve: Access helps us bend bureaucracy to work with university and external systems to create networks of support.

Ann: When we were putting together ideas for the inclusive recruitment of our researcher and project managers, access kept pushing us to think creatively about the different stages of writing the job description using language that was inclusive rather than ableist – so asking for people’s ‘experiences of …’ rather than ‘ability to perform …’

Brian: And when we came to shortlisting our applicants we understood the presence of disability not as a problem but as an opportunity. If an applicant ticks the ‘disability positive’ category and meets the essential criteria they are automatically shortlisted to the next stage.

Eve: Entering the interviewing stage of the job recruitment process, access was always present: urging us to think about creating activities, tasks and questions that were responsive to different access demands of the applicants. We also spent a lot of time giving feedback to unsuccessful disabled and non-disabled applicants. Access insisted we were transparent and informative.

la paperson frames the decolonising university ‘as an interdisciplinary, transnational, vocational university that equips its students with skills toward the applied practice of decolonisation’ (la paperson 2017, 26). This vocational inflection emphasises the job of work of depathologising the university. Access turns up in the university as a colleague, equipped with a skill set and job description. Access has work to do, conversations to start and objectives to be met. This is not to assert that matters are straightforward or easy, as the following conversations reveal.

Ann: I was accused of ‘toxic positivity’ when I argued in a department meeting that access could be read as a useful means of reworking our work culture. I think I’m understood as personifying the university’s overly positive rhetoric when I’d hope I’m doing something else!

Brian: I think the work we describe is often the opposite of positive – it feels like hard work, it’s experienced negatively and incredibly energy sapping; especially for disabled colleagues.

Eve: Could we reclaim toxic positivity? We are trying to remain positive in institutions that are fundamentally toxic; especially when we reveal their disabled and ableist constitution. Might we embrace toxic positivity; access-as-affirmation that learns from the politics of disability?

As students of critical disability studies, our default position is always critique. We have been educated to call out the university’s ableist and disablist characteristics and pathologising tendencies. And yet, as la paperson (2017, 43) tells us, we need to also ‘go through critique to get to the dirty work that might find useful machinery to further assist the decolonising university’. Depathologisation is always dirty work, not least for disabled colleagues who have to grapple every day with institutions that do not expect them to be present.

## Conclusions

This paper has drawn upon a novel composite conversations methodology to reflect on some of the ways in which we believe, we hope, we might be depathologising the university. As disabled and non-disabled principal and co-investigators of funded research projects, we are always encountering, rubbing up against, appropriating, bending and working with and through university systems, bureaucracy and policy. Our work together, with and as disabled researchers and professional services, offers opportunities to carefully, critically and often uncomfortably re-imagine research culture. We do not want to portray these narratives as success stories. We often fail and flail.

Our writing, conversations and work are being undertaken during incredibly troubling times marked by the mainstreaming of far-right politics, an unfolding economic crisis in higher education and a global backlash against EDI or DEI (Hamilton 2024; Taix and AFP 2024). To assert, to insist and, indeed, to demonstrate how the university is a depathologising university has arguably never been more urgent. While we have focused on research projects, assembling a depathologising and a decolonising university ‘is a multiscalar endeavour’ that includes ‘a single project with a decolonising aspect; a body of decolonising works, a decolonising studies …. [and] a …network of decolonising organisms, or a decolonising university movement’ (la paperson 2017, 48–49). Depathologising the university is an interdependent, affirmative and collective endeavour involving disabled and non-disabled academic, senior university and professional services colleagues, as well as disabled people’s organisations. Depathologising the university pulls in more than research projects; it means finding productive connections with colleagues in teaching and learning, staff and student support, human resources, finance, contracts, health and well-being services, trade and student unions. As Braidotti (2019a, 470) puts it, ‘the whole point of affirmation consists in inserting the practice of philosophy in such a praxis, so that we can extract from the ruins something that would—will have—triggered the inspiration to go on’.

Affirmative project leadership involves curating holding spaces and creating encounters that are engaged in the formation of research culture where we are in this together; at times in union, other times in disagreement, oftentimes in pursuit of the wider project of anti-ableist and anti-disablist in the university. Affirmation and critique are not mutually exclusive. We should never romanticise the work of depathologisation and always be open, honest and reflexive. Depathologisation is, following la paperson (2017, 43), dirty work but it is also deeply emotional, conflictual, draining, uncertain, experimental and risky labour, not least for disabled academic and professional services colleagues.

Centering disability as the driving subject of university transformation means that we engage with disability’s disruptive potential to reimagine together what it means to work in ways that engender recognition, care and consideration. It is important that we keep our conversations going and share our moments of success and, indeed, failure as part of a wider systemic project of depathologising the university. We need to get real about the university. Depathologisation demands a pause, a time to reflect and rethink the civic responsibilities of the university, that is, its accountability to all of the communities it serves. Depathologisation invites us, to meditate and reimagine the university. And those of us who work in the university are the university, and we all have work to do.

## Footnotes

1 The term learning disabilities is used in the UK, with other labels used in different countries ranging from development disabilities, intellectual disabilities and cognitive impairments. We will refrain from offering a mainstream administrative definition of learning disabilities—which would normally refer to issues of competence, intelligence and maladaptive functioning—in response to the wider aims of this paper to centralise people as researchers and analysts in their own right.
